# Quantifying Potential Error in Painting Breast Excision Specimens

**DOI:** 10.1155/2013/854234

**Published:** 2013-05-23

**Authors:** Thomas Fysh, Alex Boddy, Amy Godden

**Affiliations:** ^1^Department of Breast Surgery, South Devon Healthcare Trust, Lowes Bridge, Torquay TQ2 7AA, UK; ^2^Department of Surgery, Royal Devon and Exeter NHS Trust, Devon EX2 5DW, UK

## Abstract

*Aim.* When excision margins are close or involved following breast conserving surgery, many surgeons will attempt to reexcise the corresponding cavity margin. Margins are ascribed to breast specimens such that six faces are identifiable to the pathologist, a process that may be prone to error at several stages. *Methods.* An experimental model was designed according to stated criteria in order to answer the research question. Computer software was used to measure the surface areas of experimental surfaces to compare human-painted surfaces with experimental controls. *Results.* The variability of the hand-painted surfaces was considerable. Thirty percent of hand-painted surfaces were 20% larger or smaller than controls. The mean area of the last surface painted was significantly larger than controls (mean 58996 pixels versus 50096 pixels, CI 1477–16324, *P* = 0.014). By chance, each of the six volunteers chose to paint the deep surface last. *Conclusion*. This study is the first to attempt to quantify the extent of human error in marking imaginary boundaries on a breast excision model and suggests that humans do not make these judgements well, raising questions about the safety of targeting single margins at reexcision.

## 1. Introduction

An enduring debate amongst breast surgeons concerns the adequacy of excision margins for both invasive and *in situ* carcinoma (DCIS). As yet, no unequivocal consensus has been reached as to what exactly comprises an adequate surgical margin after breast conserving surgery (BCS) [1].

Typically, a specimen is excised and then painted or marked according to a protocol to indicate laterality and boundaries. In theory, the histopathologist receives a specimen that can then be orientated such that the location of any residual disease can be identified. It has been shown that painting specimens at the time of excision are preferable to painting by the pathology department in terms of reexcision rates [2].

While the National Institute for Health and Clinical Excellence (NICE) suggests reexcision of DCIS if the margin is closer than 2 mm, local policies vary as to what is considered an acceptable margin. Surgeons may accept a closer deep margin since the pectoralis fascia is thought to provide and robust anatomical barrier to local spread. At the Royal Devon and Exeter Hospital NHS Trust, policy is to offer reexcision to patients with any margin of invasive cancer within 2 mm and 1 mm for DCIS. Only in special circumstances will reexcision be offered for close deep or superficial margins.

It may be, however, that current practice is fundamentally flawed. The techniques described above are only valid if the six margins inked upon the excision specimen can be accurately transposed to the surgical cavity such that the area of concern can be targeted in a reexcision procedure, unless a mastectomy is chosen. This process could be compromised in several ways.

1. No breast excision specimen is a perfect cylinder or six-sided figure. The margins are in continuum such that the transition from one margin to the next is an imaginary line and must be ascribed by the person painting the specimen.
2. Given the shape of the human breast, it is easy to imagine how the shape of an excision may not actually have 6 true faces; at the edges of a hemisphere, the flat “deep” face abuts the superficial surface. Despite this, it is customary to always paint on 6 surfaces of an excision specimen.
3. The surgical cavity itself will deform over time on account of postsurgical change, radiotherapy, and surgical manipulation of the cavity.
4. The shape of the surgical cavity will vary with patient positioning and retraction during the reexcision procedure.
5. Since the cavity's margins are in continuum, the surgeon must make a subjective decision as to how large the reexcision margin should be; imaginary boundaries ascribed to the specimen must also be ascribed to the cavity.
6. Evidence exists that even when inked margins are processed in finer detail by margin shaving with multiple field examination, margin involvement is increased. Inking margins alone, therefore, is likely to underestimate margin positivity [3].

Despite these considerations, it is standard practice in most institutions to offer reexcision following BCS if margins are thought to be involved. If the reexcision specimen contains no cancer, this might be because it was all excised in the original operation, but it might also be because the wrong area of the cavity was targeted in the reexcision procedure. There is no consensus as to what the impact of reexcision is in terms of local disease recurrence, since this does not lend itself to randomised investigation. What remains in the literature is a diverse collection of retrospective, observational studies of variable robustness. Some reports suggest decreased disease-free survival in patients who have had reexcisions [4], while others report no difference [5, 6]. A further study suggested that local failure was commoner only if the reexcision specimen contained cancer [7], while another shows that local recurrence is similar when reexcision is by ink-directed cavity shaving or entire cavity excision [8]. These diverse outcomes are perhaps explained by the perpetual problem of observing local recurrence in early breast cancer; it is rare and takes many years to happen, meaning that they are usually retrospectively designed or underpowered: the latter of these studies was based on only 11 recurrence events over 3.7 years. Whatever the truth about recurrence, a second operation is undesirable in terms of delayed adjuvant treatment, operative risk, and the emotional burden this has on patients.

In this experiment, we aim to quantify, as far as possible, the error inherent in specimen painting, specifically concerning the ascription of margin boundaries and, hence, the surface area of specimen faces. This information could be used to estimate a “border of error” to be considered when re-excising cavity margins or even to question the practice altogether. We were also interested to know whether the order in which the margins were painted made a difference to the surface area ascribed as informal opinion amongst some practitioners suggested that the last surface to be painted often seemed to be larger than the rest.

## 2. Method

### 2.1. Null Hypothesis 

There is no difference in the surface areas painted on the six faces of each specimen compared to the expected calculated surface areas of the controls. 

### 2.2. The Experimental Model

An experimental model to represent a breast excision specimen was created according to the following criteria.

1. The model must be accurately replicable.
2. The model must test the ability of a person to paint, freehand, six surfaces on an irregular, nonsolid, and 3-dimensional object, using the materials they would normally use for painting breast specimens.
3. The model must be such that its painted surfaces can be scanned on a flatbed scanner.
4. The model must not be a geometrical shape with any identifiable vertices, corners, or angles.
5. The model should retain, as far as possible, the characteristics of human breast tissue (such as elasticity, malleability, and softness), acknowledging that human or animal tissue was not a suitable alternative for ethical, practical, and methodological reasons. It was also acknowledged that the model need not be a perfect proxy for breast tissue so long as it fulfilled criteria 1–4.

Having experimented with a number of fillers such as gelatine and corn flour paste, water-filled balloons were used to represent breast excision specimens, with the knot representing its most superficial aspect. It was acknowledged that the balloons could not be under any tension as this might distort the final surface area. It was found that 70 mL water achieved the desired effect.

Due to the slight convexity of the cut balloon surfaces, relaxing incisions were made after painting so that they could lie flat on the scanner. Adobe Photoshop software was used to precisely calculate the pixelated areas of interest.

Three volunteers who routinely paint breast excision specimens were asked to paint the six faces, that is, caudal, medial, cranial, lateral, deep, and superficial as if each was equal in surface area, noting the first and last surfaces to be painted. The surface areas of each aspect were measured in pixels, after being cut out (without the knot) and scanned. These measurements were compared to the expected surface area derived from a similar process whereby the balloon, unpainted, was scanned and then its total surface area divided by 6, thereby generating an almost “perfect” control in which the only variation in surface area would be due to manufacturing variability and the exact level at which the knot was cut off.

### 2.3. Statistics

The painted surface areas were compared to the control surface areas using a one-way ANOVA with Dunnett's *t*-test, a priori, multiple comparisons. Four groups of areas were compared: first painted surfaces, last painted surfaces, other painted surfaces, and control surfaces. The experimental surfaces were compared against the control surfaces with the Dunnett *t*-test. Three sample comparisons were therefore made. These were

1. first painted surfaces versus controls,
2. last painted surfaces versus controls,
3. other painted surfaces versus controls.

Statistical analysis was performed using IBM SPSS Statistics 19, and a *P* value of <0.05 was taken to be statistically significant.

## 3. Results

**Figure 1:**
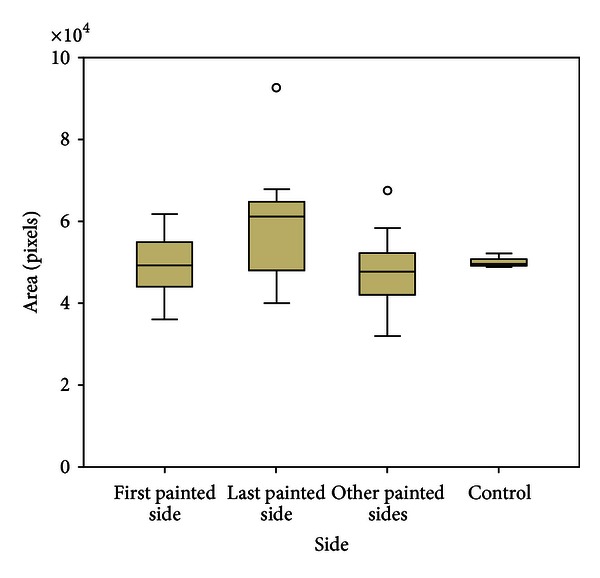
Mean surface areas and spread of painted sides and control.

**Figure 2:**
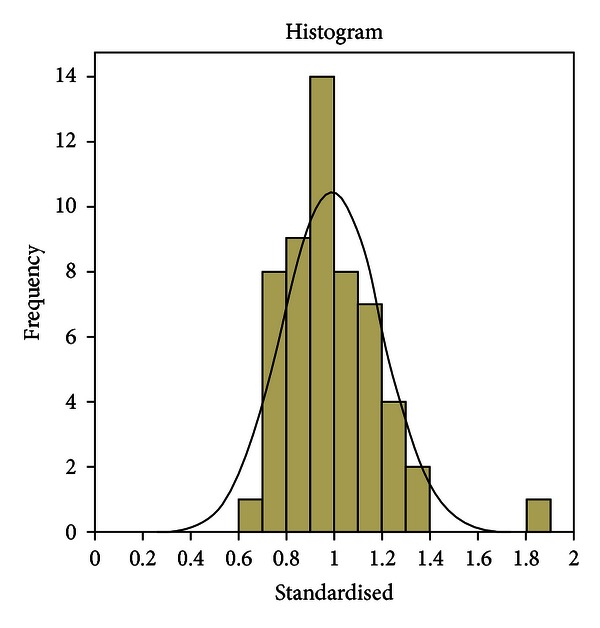
Standardised histogram (experimental surface areas divided by control mean).

Figure 1 reveals the spread observed within the experimental surfaces painted. It was not uncommon for painted surface areas to differ by as much as 100% (30 000 pixels versus 60 000 pixels), with some outliers being very much larger in variability.  Figure 2 is a standardised histogram (the experimental areas divided by the mean of the controls), which demonstrates that almost 30% of the experimental areas (16/54) were more than 20% greater or less than the control.

 Tables 1 and 2 show that no difference was observed in surface areas between the first side painted, the “other” sides painted, and the controls. There was a significant difference in the surface areas of the last sides painted and controls (mean difference 8900 pixels, CI 1477–16324, *P* = 0.014), meaning that the last side painted was on average 18% bigger than the control sides. By chance, the last side painted on every specimen was the deep margin.

## 4. Discussion

In this study, we have quantified the magnitude of error in ascribing imaginary boundaries to a breast excision model. We found enormous variation in the surface areas of the painted surfaces compared to a control and that the last painted surface was consistently 18% larger than the controls. Both of these findings raise questions as to whether painting breast excision specimens after BCS and the practice of margin reexcision (compared to cavity reexcision or mastectomy) are safe.

As with any experimental model, there are questions about translational validity, but we would submit that the kinds of judgments made by volunteers regarding boundary ascription with the balloon models are not dissimilar to those faced with a real specimen. We maintain that the problem with painting a breast specimen is not that it is breast tissue, *per se*, but that it, like the model, is irregular and has no definable shape and confluent surfaces.

The finding that some sides were frequently in the order of 100% larger than other sides is intriguing. The implications for reexcision surgery are not straightforward. For a smaller cavity margin, this might mean including a generous proportion of the surrounding cavity with the reexcision specimen, but we must acknowledge that, as the margins become bigger, the actual amounts of extra tissue are correspondingly smaller as the relationship between surface area and diameter is nonlinear and for an irregular shape would be impossible to calculate precisely.

Of similar concern is the finding that the last margin painted is consistently larger than the controls and that this was invariably the deep margin, raising the question as to whether a close “deep” margin can really be ignored as this may in fact be one of the circumferential margins.

This study is the first to attempt to quantify the extent of human error in marking imaginary boundaries on a breast excision model and suggests that humans do not make these judgements well. It might be argued that the ascription of boundaries on a specimen is only a small part of the overall problem and, given the potential areas for error, the only sensible approach is whole cavity reexcision or mastectomy. It has shown recently that an approach to BCS using oncoplastic techniques, allowing the surgeon to take larger areas of tissue whilst maintaining breast cosmesis, can result in very considerable reductions in reexcision rates [9]. Another area of interest is the development of technology to allow intraoperative assessment of margins, even before excision is complete [10] as well as devices to improve the orientation of breast specimens [11].

## Figures and Tables

**Table 1:** 

	*N*	Mean pixels	95% confidence interval	
First side	9	49053	42692	55415
Last side	9	58996	46570	71423
Other sides	36	47655	45178	56131
Control sides	24	50096	49492	50700

ANOVA, *P* = 0.003.

**Table 2:** 

	Mean difference (pixels)	P*	95% confidence interval	
First versus control	−1043	0.977	−8466	6381
Last versus control	8900	0.014	1477	16324
Others versus control	−2441	0.529	−7446	2563

*Dunnett's 2-sided *t*-test.
